# Hardware-Accelerated Non-Contact System for Sleep Disorder Monitoring and Analysis

**DOI:** 10.3390/s25092747

**Published:** 2025-04-26

**Authors:** Mangali Sravanthi, Sravan Kumar Gunturi, Mangali Chinna Chinnaiah, G. Divya Vani, Mudasar Basha, Narambhatla Janardhan, Dodde Hari Krishna, Sanjay Dubey

**Affiliations:** 1 Department of Electronics and Communication Engineering, Koneru Lakshmaiah Education Foundation, Aziz Nagar, Hyderabad 500075, Telangana, India; sravanthi.engg@gmail.com (M.S.); sravankumar.gunturi@gmail.com (S.K.G.); 2 Department of Electronics and Communication Engineering, Malla Reddy College of Engineering, Maisammaguda, Hyderabad 500100, Telangana, India; 3 Department of Electronics and Communications Engineering, B V Raju Institute of Technology, Medak (Dist), Narsapur 502313, Telangana, India; divyavani.g@bvrit.ac.in (G.D.V.); mudasar.basha@bvrit.ac.in (M.B.); harikrishna.dodde@bvrit.ac.in (D.H.K.); sanjay.dubey@bvrit.ac.in (S.D.); 4 College of Computing and Data Science (CCDS), Nanyang Technological University, Singapore 639798, Singapore; 5 Department of Mechanical Engineering, Chaitanya Bharati Institute of Technology, Gandipet, Hyderabad 500075, Telangana, India; njanardhan_mech@cbit.ac.in

**Keywords:** sleep monitoring, periodic limb movement disorder (PLMD), random forest classifier, FPGA-based accelerators

## Abstract

This study analyzes human sleep disorders using non-contact approaches. The proposed approach analyzes periodic limb movement disorder (PLMD) under sleep conditions. This was conceptualized as data capture using a non-contact approach with ultrasonic sensors. The model was designed to estimate PLMD and classify it using real-time sleep data and a machine learning-based random forest classifier. Hardware schemes play a vital role in capturing sleep data in real time using ultrasonic sensors. A field-programmable gate array (FPGA)-based accelerator for a random forest classifier was designed to analyze PLMD. This is a novel approach that aids subjects in taking further medications. Verilog HDL was used for PLMD estimation using a Xilinx Vivado 2021.1 simulation and synthesis. The proposed method was validated using a Xilinx Zynq-7000 Zed board XC7Z020-CLG484.

## 1. Introduction

Sleep disorders can significantly impact daily life, making advanced technologies crucial for achieving optimal health and well-being within the medical system. New innovations are essential for improving sleep quality and promoting a happier, healthier life. In 2004, the World Health Organization (WHO) conducted a meeting on sleep challenges [1]. According to the international classification of sleep disorders, there are over 70 distinct types of sleep-related conditions [2]. Research on identifying sleep disorders is scarce, with many existing studies concentrating on the detection of sleep apnea [3,4]. Periodic limb movement disorders are characterized by polysomnographic features that include disruptions in sleep continuity, such as arousal and awakenings [5]. Sleep disorders listed in more than 70 different PLMD challenges have been observed in approximately 4–11% of the general adult population and 5–8% of the pediatric population [5]. There are no available approaches for the early clinical analysis of PLMD. Subjects with PLMD can improve their sleep disorders by continuous monitoring. In this regard, the specialist can detect PLMD early through technological intervention [5]. Our proposed accelerator aims to analyze the different stages such as early, middle, and high-level sleep disorder, and assist doctors and patients with PLMD using a non-contact monitoring approach.

In response to the limitations of available sleep disorder detection methods, and to enable extended monitoring over periods of weeks or months, innovative methods for conducting measurements have been developed. PLMD-related data were captured from the sleep posture and movements of the population. Collecting real-time sleep data at a high resolution (every second) over the course of days for individual patients is quite challenging in standard practice. In this regard, artificial intelligence and cutting-edge deep learning technologies are essential for analyzing PLMD. This research integrated multiple challenges, such as sleep postures, periodic movement of sleep postures, static posture with lower limb movement, and data capture with non-wearable and non-contact sensors. Data mining, storage, and processing of PLMD were carried out using computing methods.

The periodic and aperiodic movements of the lower limb in sleep conditions have been estimated using different approaches, such as bed-based monitoring [6]. Bed-based sensor fusion for position changes was evaluated by Maggi and Sauter [7]. Actigraphy systems are preferred for PLMD analyses [8]. The RFID-based non-invasive method with a CNN algorithm was used to estimate sleep postures and movements [9]. Probabilistic data-driven approaches are preferred for analyzing PLMD data [10]. In [11], a trained specialist manually evaluated the Emfit (electromechanical film) sensor readings to diagnose PLMD. Although no assessment of individual events was conducted, the average number of limb movements recorded by EMG (electromyography) and Emfit demonstrated a strong correlation, with a Spearman’s coefficient of 0.87.

Sensor fusion is the preferred method used to define the PLMD details. Principal component analysis was used for the measurement fusion. The orthogonal linear transformation executed using PCA remaps the signals onto a novel coordinate framework. Within this framework, the coordinates are organized based on the eigenvalues corresponding to the newly derived eigenvectors [12]. Recent studies have presented decision fusion using a naive Bayes classifier trained during cross-validation [6]. However, effective PLMD requires computation methods. The aforementioned studies involved sensing and analysis using machine learning approaches. The real-time challenge is computing PLMD data, such as PCA, probabilistic, and naive Bayes classifiers. Cloud and edge computing approaches have been utilized, such as GPU and CPU. At the edge computing level, the replacement of a GPU with low power consumption is essential for an individual’s PLMD analysis.

This study provides an edge computing solution with an FPGA for PLMD analysis using machine learning algorithms. The novel contributions of the study are as follows:

1. A machine learning system was designed to identify conditions using sleep data collected from an unobtrusive array of non-contact ultrasonic sensors.
2. A hardware accelerator was designed for the estimation of PLMD based on a random forest classifier.
3. The subject’s PLMD was monitored using measurement fusion with FPGA-based edge computations.

This section presents the requirements and background of the PLMD analysis. This motivated us to investigate PLMD using the methods proposed in Section 2. The results of the proposed method are presented in the form of experimental validation in Section 3. Conclusions and future scope are presented in Section 4.

## 2. Methods

**Figure 1:**
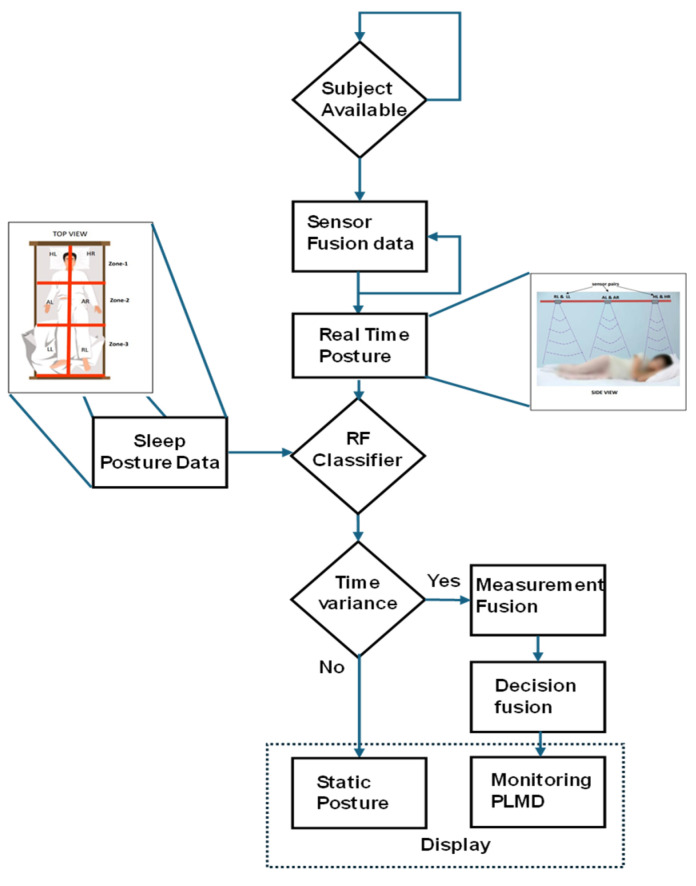
Flowchart of PLMD analysis and monitoring.

Figure 1 presents the flowchart for the movement analysis and monitoring of subjects with PLMD, with blue arrows indicating the step-by-step procedure. This approach will assist early clinical studies and healthcare professionals in monitoring the progress of PLMD treatment.

### 2.1. Machine Learning-Based Posture Analysis

Sleep postures were analyzed to determine the PLMD stage and status. In this section, data capture and machine learning analysis based on the recorded data are explained in Algorithm 1. The respective postures were classified using a random forest classifier. Sleep posture analysis was performed using training and test datasets, and the results were validated with real-time data. The proposed approach considered the sleep data reported in previous studies [13,14]. Two sets of sleep data are static and adaptive; these data were captured as echo signals converted into distance using ultrasonic sensor arrays. Xi represents a sensor feature, and its dimensions are based on the position and timing of the sensor. Sleep posture labels are represented by yi; six pairs of sleep postures were labeled as static and time-variant. D is the training dataset, and T represents the number of decision trees among the total features (M) sampled and the selected features (m). Input and initialization are the initial steps in the algorithm. Sample n instances with replacements from D at D_t and randomly select m features from M for feature selection. Tree function training was carried out with D_t and the selected features. After performing the random forest algorithm, the data were input as trained postures in F.
**Algorithm 1** Sleep Posture Classifier-Based Random Forest AlgorithmInput D={(X1,y1),(X2,y2),…,(Xn,yn)}Forest F = {} //*Initialization**for* (T = 1, T ≤ M, T + +):{D_t = bootstrap sample(D).selected features = random sample (M, m)tree = train_decision_tree (D_t, selected features)}tree. grow_fully ()forest. append (tree)F = {T1, T2, …, TT}//output the trained Random Forestreturn forest

In Line 1, we start with the input dataset D, which consists of multiple data points represented as pairs, (X1, y1), (X2, y2), …, (Xn, yn), where each Xi denotes the input features, and yi is the corresponding target value. Line 2 initializes an empty list called forest (F), which will eventually store the collection of decision trees. In Line 3, a loop is initiated to run M times, with each iteration dedicated to training one decision tree. In Line 4, a bootstrap sample D_t is created by randomly sampling data points from D with replacement, introducing variability and promoting model robustness. Line 5 explains the random selection of a subset of m features from the total set of M features, which are used for splitting nodes in the tree, ensuring diversity among the trees in the forest. In Line 6, a decision tree is trained using the bootstrap sample D_t and the selected subset of features. Line 7 ensures that the tree is grown fully without any pruning, allowing it to learn detailed patterns from the training data. In Line 8, this fully grown decision tree is appended to forest F, progressively building the ensemble. Finally, in Line 9, after all M trees have been trained and added to the forest, the complete set {T1, T2, …, TM} is returned as the final output, i.e., the fully trained random forest model.

### 2.2. Periodic Limb Movement Disorder (PLMD) Analysis and Monitoring

Algorithm 2 shows the PLMD analysis of a subject in real time is challenging; in this study, it was integrated with sleep posture and time variance.
**Algorithm 2** Periodic Limb Movement Disorder (PLMD) Analysis Algorithm{current_posture, posture_start_time} = None//*Initialization*{plmd_epi, movement_dur} = []//*Initialization**for* (i = 1, i ≤ D, i++) { 4.Xi, yi = D[i]5.  if (yi != current_posture)6.    analyze_posture (current_posture, posture_start_time, movement_dur, plmd_epi, movement_threshold)7.    movement_data = extract_limb_movement_features(Xi)} 8.if movement_data[“magnitude”] > movement_threshold:9.  movement_durations.append(movement_data[“duration”])10.if (current_posture ! = 0)11.    analyze_posture//*updated analysis*12.return plmd_epi

PLMD analysis was performed using sleep posture analysis, considering factors such as current posture and posture start time, plmd_episodes, and initialized movement duration. The analysis was performed until the loop sensor data provided the respective distances, and the results were analyzed for every iteration. Line 5 represents the respective posture, in which we trained our sleep postures from the previous algorithm with yi as the posture label, which is considered the same as the current posture. The analysis began by measuring the limb movement using fusion measurement. Movement duration is a fusion measurement parameter computed from the difference between the sensor fusion data of the previous and current limb states. Decision fusion determines the subject’s state and the status of PLMD, based on a predefined standard threshold value. However, if the movement duration parameter introduces ambiguity, it may hinder the ability of decision fusion to accurately distinguish between subjects with and without PLMD. If the presence of PLMD is confirmed, its duration also provides the PLMD stages.

### 2.3. Hardware-Based Accelerator for Posture Analysis

**Figure 2:**
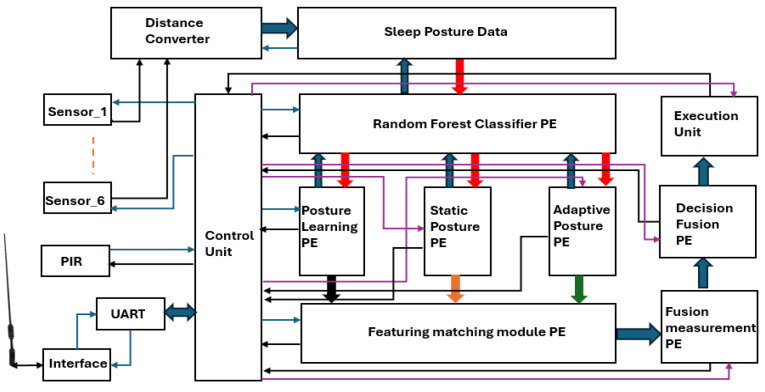
The overall architecture of PLMD analysis and monitoring.

The analysis and monitoring of PLMD with a VLSI architecture are presented in Figure 2.

The analysis was performed using sleep data capture and distance normalization. A posture-learning processing element (PE) using a random forest classifier (RFC) processing element was employed for learning updates based on the captured sleep data. Current posture was registered using static and adaptive PEs, and the real-time sleep postures were compared with the learned postures for matching. Limb movements were measured using the fusion measurement PE, and the stage of PLMD was confirmed by the decision fusion PE. The decision was displayed and communicated through the execution unit. The control unit operated at 100 MHz and controlled all processing elements.

#### Data Capture and Normalization

**Figure 3:**
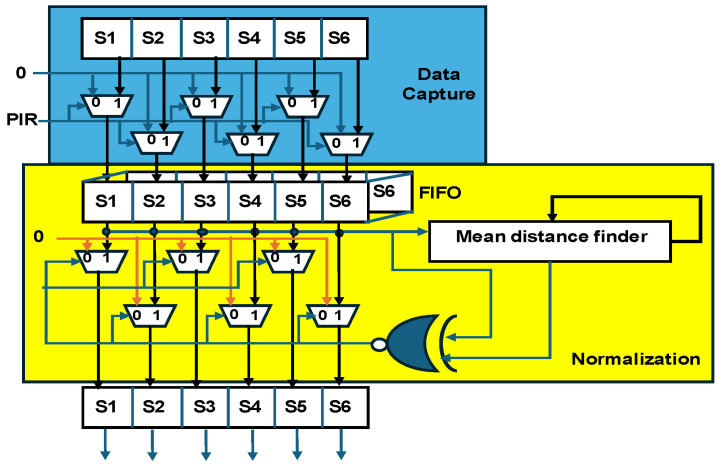
Hardware scheme for data capture and normalization.

Figure 3 shows the hardware scheme for data capture and normalization. In this study, data were collected using six pairs of ultrasonic sensors, with the readings stored in arrays S1–S6. Data collection was enabled using a PIR sensor, which triggered the recording process, and all the external interfaces with the control unit are represented by blue lines. The FIFO buffer is defined with dimensions L × W × H, where L (length) = 20 bits, W (width) = 6 arrays, and H (height) = 40. The height value corresponds to the data stored at four iterations per second for 10 min (4 iterations × 10 min = 40 data blocks).

The mean distance finder plays a key role in the normalization process. Normalization was parameterized using two values, namely early normalization and encoded normalization, which correspond to different physical conditions of the subject. Early normalization refers to distance computation in the absence of a subject and may vary depending on time and environmental conditions. Encoded normalization, on the other hand, is based on trained sensor data specific to the subject. The mean distance finder processes both normalization values and provides feedback by comparing the previous mean distance with the current mean distance, enabling precise normalization across multiple data iterations.

### 2.4. Hardware-Based Accelerator for PLMD Analysis and Monitoring

#### 2.4.1. Random Forest-Based Fusion Decision

**Figure 4:**
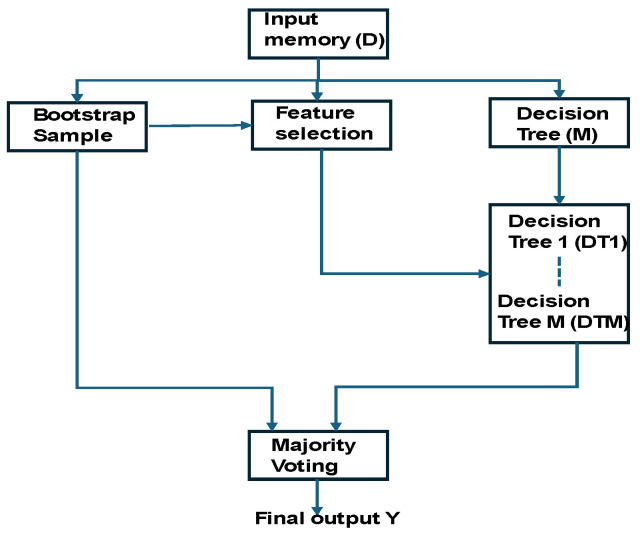
Flowchart of random forest-based fusion decision (RFFD).

The random forest-based fusion decision (RFFD) flowchart is presented in Figure 4. Sensory data (D) were captured and stored in the input memory. This input was then shared with the bootstrap sampling, feature selection, and decision tree construction modules (denoted as M). The bootstrap samples were directed to both majority voting and feature selection processes. A series of decision trees were constructed up to M, and each tree was integrated with its corresponding selected features. Posture features were selected based on posture labels, such as supine pose, covering both static and movement postures. The decision trees were constructed using the random forest method, and the number of iterations (m) was determined based on the accuracy results. The final fusion decision was established through a majority voting system across the ensemble of trees.

**Figure 5:**
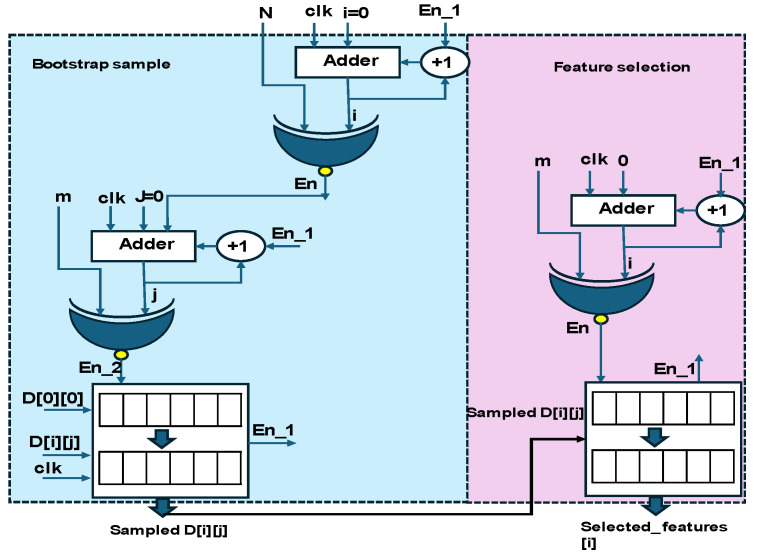
Hardware schemes of bootstrap sample modules and feature selection.

The bootstrap sample and feature selection hardware schemes are shown in Figure 5. They were processed based on two iterations and resulted in the sampled data D[i][j]. The resulting data were fetched into the feature selection module. The features of the pose were considered from the standard posture methods [13]. In digital models, the binary array patterns derived from data capture and normalization were used as references. The selected features [i] were fed to the RF decision tree.

**Figure 6:**
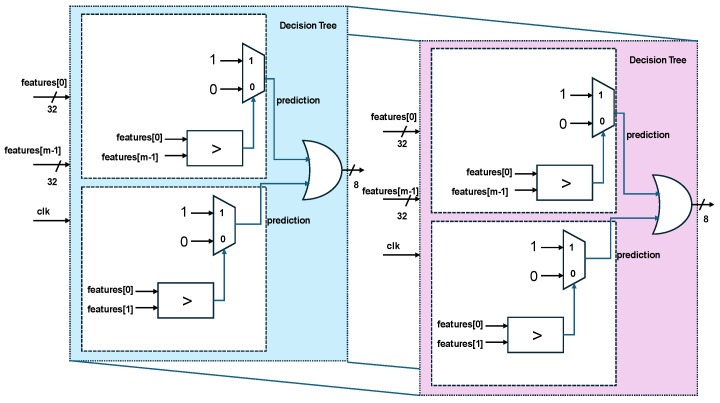
Hardware schemes of random forest-based decision tree.

The RF decision tree consists of a number of submodules based on accuracy and consists of the features selected through comparisons, as shown in Figure 6.

**Figure 7:**
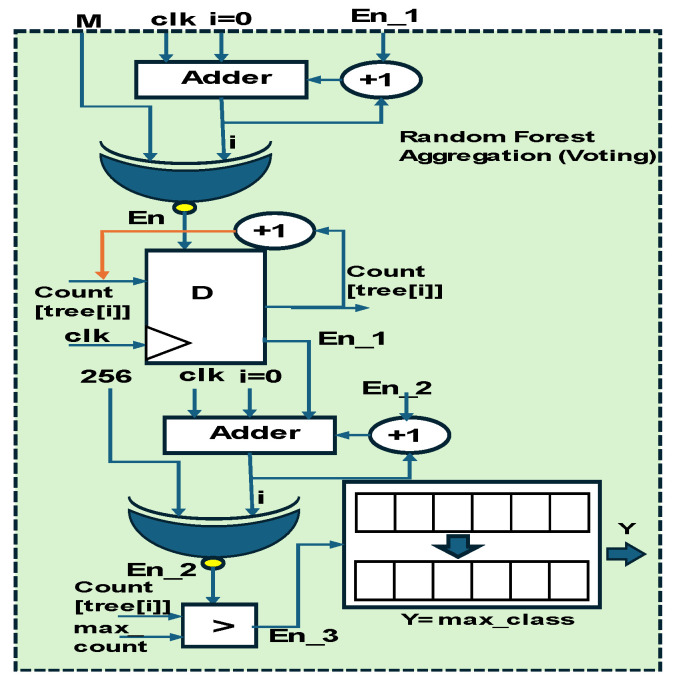
Hardware scheme of random forest voting.

The final stage of the RF modules is voting, as shown in Figure 7. This is initiated by storing the count values, which are compared with the maximum value; the Y output is retained as the max class.

#### 2.4.2. Fusion Measurement Based on Time Variance

**Figure 8:**
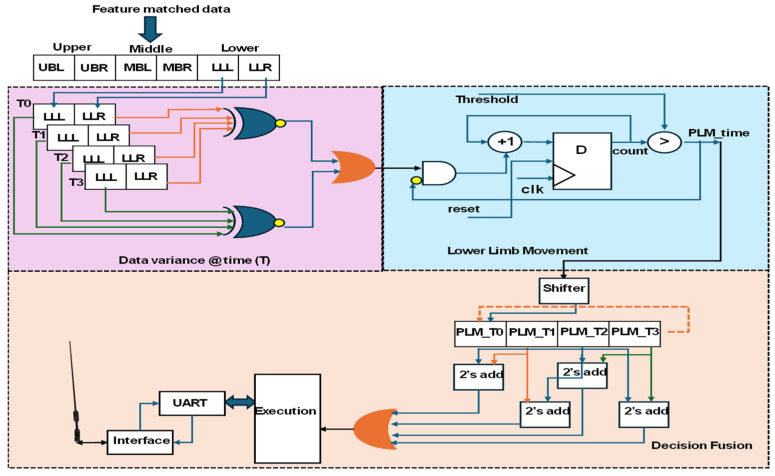
Hardware schemes of fusion measurement and decision fusion.

This module aims to define the subject positioned in a static or adaptive posture and detect PLMD using the feature-matching PE, the decision fusion PE, and the fusion measurement PE. As shown in Figure 4, in the RF classifier, a static posture is considered a time-invariant posture as per standard [13], and subject movement from point to point within the sleeping area represents an adaptive posture, as defined in [14]. The feature-matching module PE was constructed using a binary search tree [15], which plays a vital role in fusion measurement and decision fusion PEs. The core of the PLMD analysis involves hardware schemes, as shown in Figure 8.

The feature-matching processing element (PE) provided posture confirmation, which served as input to the fusion measurement PE. The integrated data variance over time, in both static posture and lower limb movement, was used to evaluate PLM (periodic limb movement) duration. Data variance was captured at four iterations per second and stored over T1 to T3 time intervals. The lower limb—left (LLL) and lower limb—right (LLR) readings were compared each second to detect variations. When limb movement was detected, a duration counting module was activated, with thresholds set based on the subject’s PLMD stage. The duration values were stored in a FIFO buffer in the decision fusion block using a circular shifter mechanism. By comparing adjacent time windows, the system identified the duration of limb movement and inferred the subject’s PLMD stage. Before clinical diagnosis, the execution module shares this information with either a doctor or the subject’s caregiver, enabling a better understanding of PLMD.

## 3. Results

The results of the proposed PLMD monitoring method using hardware schemes are presented in this section.

### 3.1. Experimental Setup

**Figure 9:**
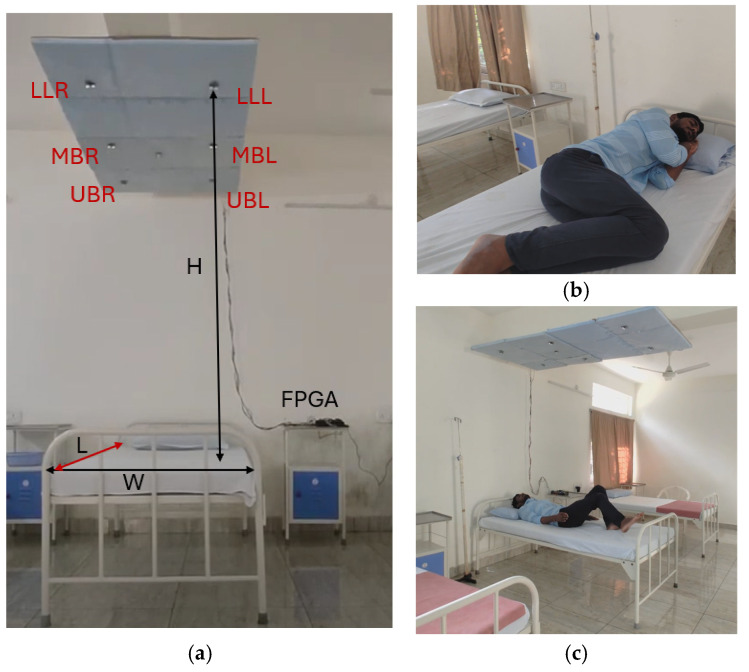
(**a**–**c**) Illustration of the experimental setup of contactless sleep posture analysis.

The PLMD monitoring experimental setup, shown in Figure 9a–c, comprised ultrasonic sensors for sleep data capture and a PIR sensor for the estimation of the availability of bed space. These sensors were positioned on the ceiling above the bed, facing towards it. Data processing and PLMD detection were carried out using a field-programmable gate array device (FPGA). Six pairs of PWM-based ultrasonic sensors (manufactured by Rhydo Labz, India) were used to capture real-time sleep posture data.

**Figure 10:**
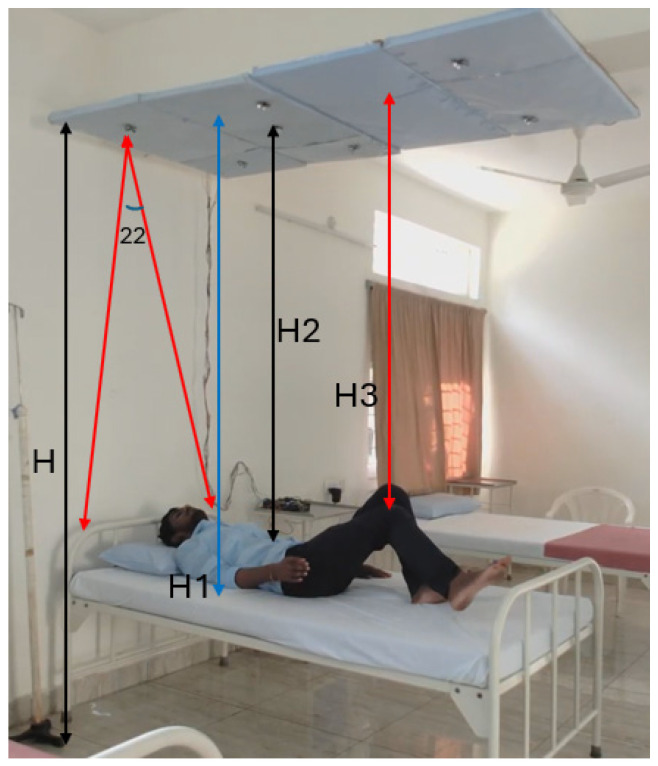
Illustration of the experimental setup for analysis with sensors placed at a distance.

Ultrasonic sensors operated at a frequency of 40 kHz with a 5 V DC supply, consuming less than 20 mA of power. An ultrasonic sensor produces echoes and converts them into distances using the pulse width modulation technique. The sensors were precisely positioned to detect distances ranging from 2 cm to 4 m, providing stable, non-contact measurement data essential for accurate posture analysis. The complete analysis of experiment is shown in Figure 10.

Figure 9a represents the environmental setup for the experiment. The distance between the sensor array and the bed was recorded as the height (H), the distance between the front and end of the bed was recorded as the length (L), and the width (W) was recorded as the difference between the left and right edges of the bed. Sensor arrays were positioned at the upper side (UBR, UBL), middle (MBR, MBL), and lower side (LLL, LLR). The distance was captured every 0.25 s from all sensors concurrently and processed using the hardware schemes. Machine learning was performed using the sensor’s distance and the time variance between distances for PLMD detection.

In this study, the difference between the ceiling and floor (H) was 2.8 m, and the distance between the sensor array and the bed (H_1_) was 2.2 m. The ultrasonic sensor operated with a coverage of 22°; the area covered on the bed with an H_1_ distance was around 80 cm. H_2_ was 2.1 m, and H_3_ was 1.9 m.

Distance covered on bed = Tan (22°) × 2.2 m (H_1_ distance) = 88 cm. = Tan (22°) × 2.1 m = 84 cm. = Tan (22°) × 1.9 m = 76 cm.(1)

The H_3_ distance refers to the area covered on the bed (76 cm). In this regard, lower limb movement at H_3_ was recorded with event-driven conditions every 0.25 s.

### 3.2. Resource Utilization

The FPGA Xilinx Zynq-7000 Zed board was used for computing the proposed PLMD monitoring algorithms. Table 1 presents an outline of resource consumption for PLMD analysis and monitoring using hardware schemes. A Xilinx reconfigurable device (San Jose, CA, USA) was used, which is part of the Xilinx Zynq family, namely an XC7Z020-1CSG484 Zed board. In this approach, a processing system (PS) was used to record time-stamped tasks during service execution. The Zed board’s programming logic (PL) is responsible for controlling the logic and interfacing. To achieve system-level synchronization, the PS and PL were integrated using the AXI lite protocol. The Zed board includes block RAM (BRAM), consisting of 140 blocks (36 kb, equivalent to 4.9 Mb) and 220 digital signal processing (DSP) slices, which were partially employed in this method. The operated frequency of FPGA was 100 MHz, and it was synchronized with the external components using UART communication modules and PWDC modules for ultrasonic sensors.

Among the various computing approaches, edge computation is the most efficient and accurate. FPGA-reconfigurable devices excel in power efficiency and parallel processing capabilities [16]. In the field of PLMD analysis, FPGAs are at the forefront of providing adaptive position–event solutions with respect to time variance. The resource utilization of FPGAs was evaluated using three metrics: lookup table (LUT), block RAM (BRAM), and digital signal processing (DSP) slices.

**Figure 11:**
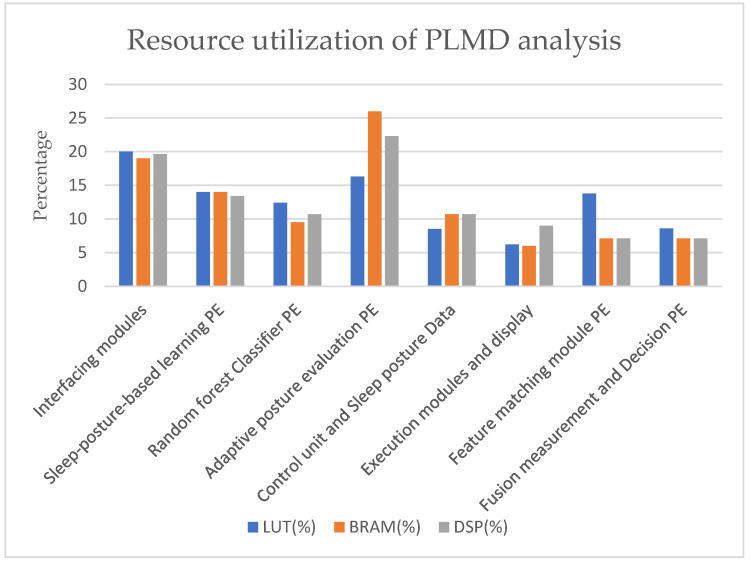
Resource utilization of PLMD analysis accelerator.

The overall resource consumption was as follows: 59% of LUT, 60% of BRAM (2.94 Mb), and 51% of DSP slices. The resource utilization of each module is presented in Figure 11. The x-axis represents the computational modules, and the y-axis shows the percentage of resource utilization. The interfacing modules consumed 20% of the LUT, 19% of the BRAM, and 20% of the DSP slices of overall device utilization, indicating more LUT consumption in these modules. The adaptive posture PE consumed the most BRAM and DSP slices, around 26% and 22%, respectively. It also involved more traversing steps and data storage. It is a key player in PLMD feature measurement.

**Figure 12:**
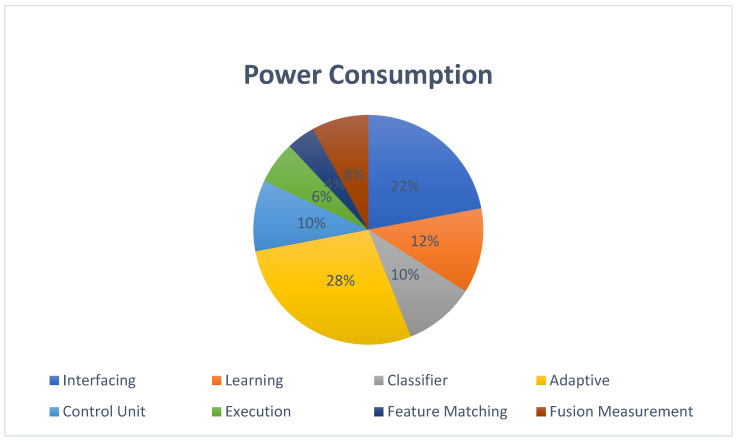
Device power consumption of PLMD analysis accelerator.

The power consumption of the device when operating a reconfigurable device (FPGA) is shown in Figure 12. According to the Xilinx power estimator (XPE), the static device power consumption was 1.4 W. In the overall power distribution, the power consumption of the adaptive posture PE accounted for 28%. XPE analysis also provided power consumption data for the other components. The second-highest power demand was attributed to interfacing with the external modules.

The total power consumption of the device was 1.4 watts, comprising 0.98 watts of dynamic power and 0.42 watts of static power. The hardware configuration employed 12 pipeline stages (S) and operated at a device clock time (Tclk) of 10 ns. The validation process involved 40 (N) iterations. Equations (2) and (3) describe the 510 ns latency observed in the proposed hardware scheme.

Latency per iteration = 12 × 10 ns = 120 ns(2)

Total latency = (N + S − 1) × T clk = (40 + 12 − 1) × 10 ns = 510 ns.(3)

The system demonstrated an overall accuracy rate of 97.5%. This figure was calculated by combining the data from multiple sensors. The system successfully made 40 and 39 accurate data predictions. The accuracy and error rate calculations are shown in Equations (4) and (5), respectively. As indicated in Equations (6) and (7), the system achieved an accuracy rate of 97.5% with a corresponding error rate of 2.5%.

(4) $$Accuracy=\frac{Number\;of\;Correct\;Predictions}{Total\;Predictions}\times 100$$

Error rate = 1 − Accuracy(5)

(6) $$Accuracy=\frac{39}{40}\times 100=97.5\%$$

(7) $$Error\;rate=1-\frac{39}{40}\times 100=2.5\%$$

### 3.3. Experimental Results

**Figure 13:**
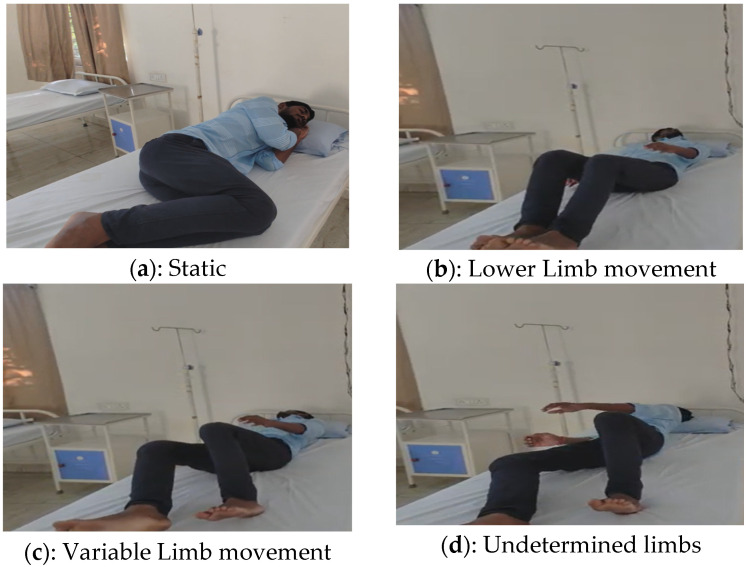

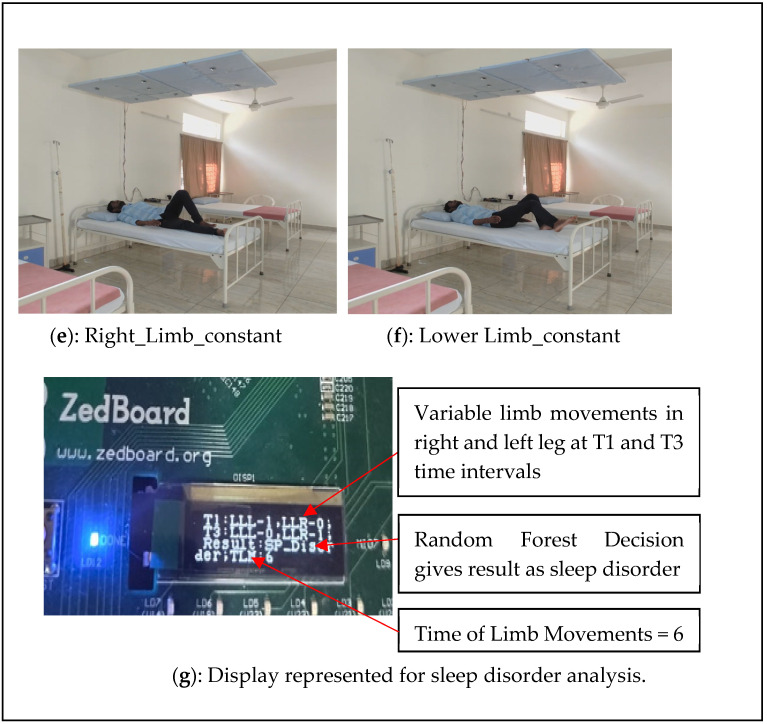
(**a**–**g**) Experiment results of PLMD with a volunteer.

The experimental validations are represented in Figure 13a–g. The PLMD experimental results are shown in Figure 13a–f. The findings indicate that the proposed hardware schemes are versatile for the detection of PLMD. In Figure 13a, the subject is positioned in a static posture facing toward the right in the fetal pose. After some time in the supine position, lower limb movements were observed (Figure 13b,c). Figure 13d shows the undetermined states of the limbs due to continuous variable limb movements. PLMD versatility was observed with constant right limb movements, as shown in Figure 13e. Figure 13f represents constant movements in the right and left limbs. All the sleep postures were analyzed continuously using the FPGA accelerator. The determined sleep disorder is shown in Figure 13g for the postures shown in Figure 13c–d. Posture versus limb movements with respect to the period were classified as both-limb and one-limb movements, and both were included in the analysis. The PLMD analysis, displayed on the LCD of the Zed board FPGA, is illustrated in Figure 13g, presenting an alternative approach to limb movement analysis. In this experiment, T1 marks the initial event where the lower limb—left (LLL) is in motion, represented as logic ‘1’, while the lower limb—right (LLR) remains static, denoted as logic ‘0’. At event T3, the LLL becomes static (logic ‘0’), and the LLR is in motion (logic ‘1’). The time of limb movement (TLM), representing the duration of limb activity, was measured as six units. Each unit duration may vary depending on the individual’s condition or PLMD stage.

PLMD was detected with four different lower limb movements: In Exp1, both left and right limbs moved. In Exp2, the right limb remained constant, whereas the left limb moved. In Exp3, only the right lower limb moved. In Exp4, limb movement was constant across various durations; thus, it was classified as a non-PLMD status. When both limbs changed position for only one instant and then maintained a static position, it was considered a pose change and therefore not PLMD. The x-axis represents lower limb movement events, including static, as well as PLMD in the left, right, and both limbs, while the y-axis shows the number of limb movements per unit time.

**Figure 14:**
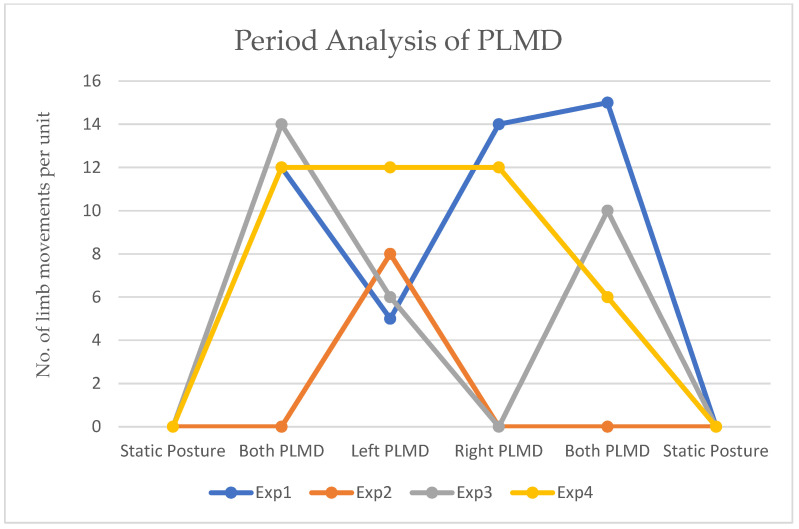
Experiment results of PLMD analysis with respect to periods.

Figure 14 shows the experimental results when new findings were observed. The x-axis is labeled as the event type, and the y-axis counts the number of movement units. PLMD patients displayed limb movements in different forms. In Exp1, subjects presented both limb movements with 12 and 15 units, and individual limb movements were observed, with 5 units for the left limb and 14 units for the right. In Exp2, only left lower limb movements were recorded, with eight units. As mentioned in the above accuracy analysis, the offset values from static to limb movements were not clear, and the error rate was approximately 2.5% in this case. Similarly, movements in both limbs and the left limb alone were observed in Exp3. Each unit was considered based on velocity, step size, and step size from the initial pose to another pose with respect to limb movements as computed using the distances from the ultrasonic sensors. In Exp4, limb movement was constant across various durations and therefore was classified as non-PLMD status. The limitation of early-stage PLMD analysis is that small movements with respect to step size are challenging to analyze, as is the very small step size at the beginning of limb movements.

### 3.4. Comparison

The proposed hardware schemes for PLMD detection are compared with relevant research methods in Table 2. Novel hardware schemes were developed for the identification and monitoring of PLMD. Non-wearable approaches have been developed with 97.5% accuracy and the utilization of machine learning algorithms for analysis. FPGA is advantageous in this approach for the concurrent computation of algorithms with synchronization.

## 4. Conclusions

In this study, PLMD detection and its challenges were investigated using hardware accelerators. The proposed approach involves data capture and normalization using non-wearable sensors and hardware schemes, the results of which were inputted into the accelerators. PLMD monitoring and analysis were performed using a hardware accelerator with a random forest classifier and binary-based feature-matching algorithms. These tools proved effective, with an accuracy of 97.5% and optimized power consumption of around 1.4 watts for computation. The limitation is that the analysis of early stages of PLMD involves small movements with respect to step size. Future research should focus on analyzing early movements with small step sizes and velocity. Partial reconfiguration is another possible future method for analyzing multiple scenarios.

## Figures and Tables

**Table 1:** FPGA resource utilization for PLMD analysis accelerator.

Module	LUT	BRAM	DSP Slices
Interfacing modules (sensors, communication (UART), Xilinx IP cores)	6362	16	22
Sleep-posture-based learning PE	4404	12	15
Random forest classifier PE	3916	8	12
Adaptive posture PE	5140	22	25
Control unit and sleep posture data	2692	9	12
Execution modules and display	1958	5	10
Feature-matching PE	4342	6	08
Fusion measurement and decision tree PE	2714	6	08
Total	31,528	84	112

**Table 2:** Comparison of sleep posture analysis with relevant research methods.

Reference Paper	Sensory Approach		Algorithm	Hardware	Pros	Accuracy	Cons
	Method	Fusion					
Waltisberg et al. [6]	Pressure sensors	Yes	PCA and CNN	GPU	Classification sensitivity 56.2%	specificity of 86.0%	Contact approach
X. Hu et al. [9]	RFID	Yes	CNN, k-means	CPU	Body movements	86.284%	Increased usage of sensors
S. Kolappan et al. [17]	Wearable sensors	Yes	NN, SDNN	GPU	Health monitoring	N/A	Limited Analysis
S. Kye et al. [18]	Motion sensors	Yes	SVM, Random Forest	Wearable	Health monitoring	96.92%	N/A
Proposed	6 Ultrasonic sensors	Yes	Random forest classifier, Binary search Feature Matching	FPGA	Parallel computing, <370 ns, sampling, and computation.	97.5%	Early glitches @ PLMD

## Data Availability

The original contributions presented in this study are included in the article and available at: https://github.com/Chinnaiah21/Ultrasonic-sensor-data. Further inquiries can be directed to the corresponding author.

## Abbreviations

The following abbreviations are used in this manuscript:

PLMD	Periodic limb movement disorder
FPGA	Field-programmable gate array
GPU	Graphics Processing Unit
CNN	Convolution neural network
Xi	Sensor Feature, considering units in centimeters
yi	Sleep posture labels, considering in seconds
D	Training dataset
T	Number of decision trees
M	Total Features, considering units in seconds
m	Sampled with features
D_t	Bootstrap sample (D), per second
LLL	Lower limb—left
LLR	Lower limb—right
UBR	Upper-bed, right-side
UBL	Upper-bed, left-side
MBR	Middle-bed, right-side
MBL	Middle-bed left-side
LLL	Lower limb—left
LLR	Lower limb—right
TLM	Time of limb movement
UART	Universal asynchronous receiver/transmitter
RFID	Radiofrequency identification
RFFD	Random forest-based fusion decision
RF	Random forest
